# Gene structure of the pregnancy-associated glycoprotein-like (PAG-L) in the Eurasian beaver (*Castor fiber* L.)

**DOI:** 10.1007/s10142-017-0557-9

**Published:** 2017-03-29

**Authors:** Aleksandra Lipka, Marta Majewska, Grzegorz Panasiewicz, Martyna Bieniek-Kobuszewska, Bozena Szafranska

**Affiliations:** 1 Department of Animal Physiology, Faculty of Biology and Biotechnology, University of Warmia and Mazury in Olsztyn, Oczapowskiego Str 1A, 10-719 Olsztyn-Kortowo, Poland; 2 Department of Gynecology and Obstetrics, Faculty of Medical Sciences, University of Warmia and Mazury in Olsztyn, Niepodleglosci Str 44, 10-045 Olsztyn, Poland; 3 Department of Human Physiology, Faculty of Medical Sciences, University of Warmia and Mazury in Olsztyn, Warszawska Str 30, 10-082 Olsztyn, Poland

**Keywords:** APs, Aspartic proteinases, Beaver, PAGs, SNVs, Exon-intron structure

## Abstract

The pregnancy-associated glycoprotein-like family (PAG-L) is a large group of chorionic products, expressed in the pre-placental trophoblast and later in the post-implantational chorionic epithelium, and are involved in proper placenta development and embryo-maternal interaction in eutherians. This study describes identification of the PAG-L family in the genome of the Eurasian beaver (*Castor fiber* L.), named CfPAG-L. We identified 7657 bp of the *CfPAG-L* gDNA sequence (Acc. No. KX377932), encompassing nine exons (1–9) and eight introns (A–H). The length of the *CfPAG-L* exons (59–200 bp) was equivalently similar to the only known counterparts of *bPAG1*, *bPAG2*, and *pPAG2*. The length of the *CfPAG-L* introns ranged 288–1937 bp and was completely different from previously known *PAG* introns. The exonic *CfPAG-L* regions revealed 50.3–72.9% homology with equivalent segments of *bPAG1* and *pPAG2* structure. The intronic *CfPAG-L* regions alignments revealed a lack of homology. Within the entire *CfPAG-L* gene, 31 potential single nucleotide variants (SNV: 7 transversions and 24 transitions) were predicted. The identified exonic polymorphic loci did not affect the amino acid sequence of the CfPAG-L polypeptide precursor. This is the first report describing the *CfPAG-L* gene sequence, structural organization, and SNVs in the Eurasian beaver, one of the largest rodents.

## Introduction

Since the Eurasian beaver is not a common subject of the scientific studies, the number of papers on this species is very limited. Within the Rodentia order, the Castoridae family is represented by only two still extant species, *Castor canadensis* in North America and *Castor fiber* (Cf) in Eurasia (http://www.iucnredlist.org/details/4007/0). Both species have a similar appearance and can be distinguished only by cytogenetic analyses indicating 40 or 48 chromosomes in the American and the Eurasian beaver, respectively (Lavrov and Orlov 1973). However, many aspects of the physiological knowledge of beavers still remains completely unknown, especially reproduction and pregnancy. Limited data originate from difficulties with tissue sampling of this taxon. Previous multi-gene studies have suggested Geomyoidea taxa to be the closest relatives of the beavers (Montgelard et al. 2008; Blanga-Kanfi et al. 2009). But, more recent molecular data strongly support the placement of *Castor* within a “mouse-related clade” with several families, including Pedetidae, Anomaluridae, Muridae, Dipodidae, Geomyidae, and Heteromyidae (Horn et al. 2011).

Genomic studies of most mammals to date have revealed a predominance of multi-gene families whose products are expressed in some reproductive organs. Within the placenta, the chorionic trophoblast constitutes the outer embryo-derived cells that form an essential interface between the maternal uterus and the embryo-originated placental membranes (Wallace et al. 2015).

Pregnancy-associated glycoproteins (PAGs) belong to the multigenic aspartic proteinase (AP) family, widely distributed in various taxa, which also includes pepsins (A, C and F), cathepsins (D and E), and various other enzymes as plasmepsins or napsins (see Szafranska et al. 2006). All members of the APs, possess a two-bilobe configuration with a cleft (with two Asp residues within two domains), capable of binding short peptides. Pepsins accomplish digestive functions outside the cell, whereas cathepsin D and E are typical intracellular zymogens, generally localized in the lysosomal compartment that provides the acidic environment necessary to accomplish their catalytic function (Kageyama 2002; Carginale et al. 2004). Interestingly, the PAG-L family products function as various chorionic signaling ligands interacting with gonadal and extra-gonadal gonadotropin receptors of cyclic pigs and cows (Szafranska et al. 2007) or early pregnant pigs (Panasiewicz et al. 2007).

To date, the entire exon-intron organization structures have been identified for only three genes: bovine *PAG1* – *bPAG1* (Xie et al. 1995), porcine *PAG2* – *pPAG2* (Szafranska et al. 2001), and *bPAG2* (Telugu et al. 2009). The mammalian *PAGs* and many alternatively named *PAG-Like* (*PAG-Ls*) are the most closely related to the pepsin family. The identified *PAGs* possess a conserved structure that includes nine exons and eight introns (A–H), among which the intron F is the longest in *pPAG2*, *bPAG1* and *2* (Wallace et al. 2015; Bieniek-Kobuszewska et al. 2016). However, the organizational structures of the *PAGs* have not been studied and, therefore, remain completely unknown in the genomes of the Rodentia taxa.

In our previous studies, we identified 1257 bp of the *Castor fiber* PAG-Like (*CfPAG-L*; KU245742) cDNA sequence, encoding 391 amino acid (aa) of entire polypeptide precursor, composed of 16 aa signal peptide, 46 aa pro-piece, and 329 aa of the mature protein, with one site of potential N-glycosylation and two Asp residues specific for the catalytic cleft of APs (A. Lipka et al. unpublished). In addition, among the diversified cellular and secretory CfPAG-L profiles, we identified dominant 58 kDa isoform, which was immuno-detected despite the fetus sex and the multiplicity of gestation. The CfPAG-L expression was localized within mono-nucleated and giant trophectodermal cells of the beaver discoidal placenta (A. Lipka et al. unpublished). The identified characteristics of the CfPAG-L family (placental cDNA encoding polypeptide precursor; also the cellular and secretory proteins) should be complemented by a genomic analysis. Thus, the aim of this study was identification and broad-based characterization of the *CfPAG-L* with its exon-intron structure and a potential polymorphism—single nucleotide variants (SNVs) in the genome of the Eurasian beaver, originating from the Polish population.

## Materials and methods

Beavers were captured and euthanized with government permits from the Regional Directorate for Environmental Protection in Olsztyn (RDOS-28-OOP-6631-0007-638/09/10/pj), and the III Local Ethical Commission for Experiments on Animals at the Warsaw University of Life Sciences (11/2010), confirmed by the Local Ethical Commission for Experiments on Animals at the University of Warmia and Mazury in Olsztyn (UWM/111/2011/DTN).

Blood samples were harvested post mortem from the jugular veins of male and female beavers (*N* = 15). Collected samples were transported in ice to the laboratory and centrifuged (3.500×*g*) for 30 min at 4 °C. Plasma was discarded and the buffy coats of white cells were separated from red cells and immediately stored at −70 °C until genomic DNA isolation.

### Identification of the PAG-L gene in the beaver genome

Leukocyte samples of 15 beavers: 5 females (pregnant); 5 males (potential fathers of the offspring) and 5 fetuses; were used as a source of DNA. Genomic DNA (gDNA) templates of Cf were isolated from leukocytes with the use of a commercial available kit (Sherlock AX, A&A Biotechnology, Poland). Only high-quality gDNA templates were used for PCR amplifications (700 ng) of the exonic and/or intronic *CfPAG-L* fragments. In order to identify the initial and partial nucleotide sequence of the *CfPAG-L*, the gDNA amplicons were produced with homological primers (Table 1), designed on the *CfPAG-L* cDNA sequence identified previously (KU245742). For effective multiple PCRs, JumpStart™ Taq ReadyMix™ (Sigma-Aldrich) was used for amplifications under the following conditions: initial activation (95 °C/2 min), then 40 cycles (95 °C/1 min for the denaturation of gDNA templates; 60 °C/1 min for primer annealing; and 72 °C/4,5 min for amplicon synthesis). Obtained amplicons: *CfPAG-L* gDNA, porcine *PAG10* cDNA—used as a positive control, and negative control (without templates)—were separated in 1% agarose gels, parallel to a marker (100–3000 bp; Thermo Fisher Scientific, USA), UV-visualized using Midori Green Nucleic Acid Staining Solution (NIPPON Genetics Europe GmbH, Germany) and then archived (G:Box, Syngene, UK).

**Table 1:** Specific primers applied for the *CfPAG-L* amplifications with gDNA templates

Forward primers		Reverse primers	
Name	Sequence (5′–3′)	Name	Sequence (5′–3′)
ATG_F	ATGAAGTGGATAGTGGTGGCC		
Exon2_F	TTCCTGAAGAMSCACAA	Exon2_R	TCATGGAGAACACTGAAGTC
Exon3_F	GCTCCTCCAACCTGTGGGT	Exon3_R	ACCCACAGGTTGGAGGAGCC
Exon4_F	ACCTACCACACCGATAAGAAGA	Exon4_R	TCTTCTTATCGGTGTGGTAGGT
Exon5_F	GATGGCATCMTGGGSCTGGCCTA	Exon5_R	TAGGCCAGSCCCAKGATGCCATC
Exon6_F	GTGTGGACAAGCGCCTGTACAA	Exon6_R	CTCCCCCATCCTTAGAGCCTTC
Exon7_F	TTGTKGACACMGGCACCTCTCTG	Exon7_R	CAGAGAGGTGCCKGTGTCMACAA
Exon8_F	CTGCCCACACTCGACTTCATCA	Exon8_R	TGATGAAGTCGAGTGTGGGCAG
Exon9_F	ACTGCATGGTGGGAATCCAG	Exon9_R	GAAGACATCWCCMAGGATCCAA
IntronA_F	GCAGCCCTTGAATGACAGGTAT	IntronA_R	CCTCCTTTTTCAGTATGAGTGCAA
IntronB_F	CTTGTGAGCTAACAGCCTGCC	IntronA_R2	TGGCTCTCACTTACAATCTCCATG
IntronD_F	GCAAGGTTGAAGAATGAGCCTAG	IntronA_R3	TTCCTCCATGACACCAACAAAGAC
IntronF_F	CCACAGGTGTCCTCTGCATGA	IntronE_R	TTCGGGTTTCATGTAGAGTGAG
IntronG_F	GGGTTAGAGCAAAATTACTGGAAC	3’utr_R	CCAGAGAAGAGGCACAGATAGA

Electrophoresed *CfPAG-L* gDNA amplicons were gel-out purified and used as templates for capillary sequencing (3130 Genetic Analyzer, Applied Biosystems, USA) in sense and anti-sense directions. The gDNA amplicons labeling was performed with a BigDye Terminator v3.1 Cycle Sequencing Kit (Applied Biosystems, USA), but with some modifications to the original manufacturer procedure. Briefly, the labeling conditions were: initial denaturation (at 96 °C for 1 min), and then 30 cycles (96 °C/10 s, 50 °C/5 s, 60 °C/4 min). Each labeling mix (20 μl) contained: 12 μl (5–10 ng) of amplicon templates, 1.2 μl Ready Reaction Mix, 4 μl BigDye Terminator v1.1/3.1 Sequencing buffer (5×), 2 μl of primer and 0.8 μl H_2_O. The labeled gDNA amplicons were purified with the BigDye X Terminator Purification Kit and separated in capillaries filled with POP-7™ polymer (Applied Biosystems, USA). The obtained *CfPAG-L* sequence data were analyzed by Geneious R7 software (http://www.geneious.com, Kearse et al. 2012). Exon-intron structure was predicted (http://www.cbs.dtu.dk/services/NetGene2/) and then confirmed by comparison of the entire *CfPAG-L* gDNA sequence with previously identified *CfPAG-L* cDNA (KU245742).

## Results

**Fig. 1:**
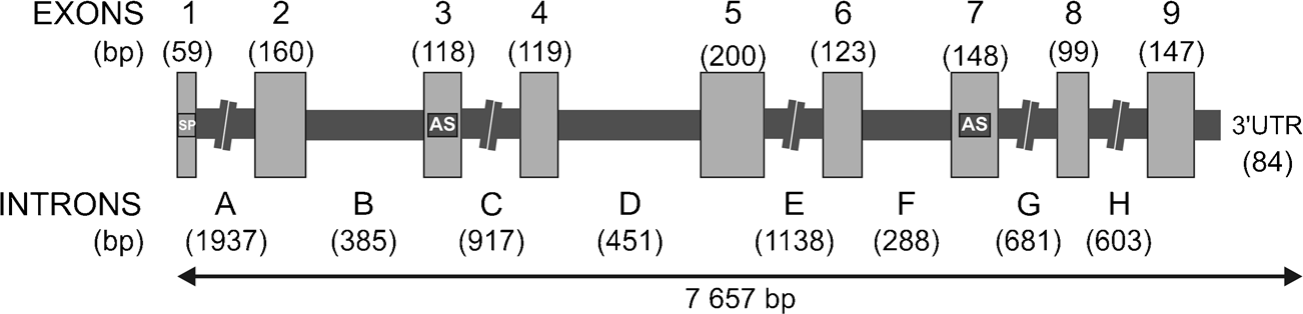
Exon-intron structure of the identified genomic *CfPAG-L* sequence. Abbreviations: *SP* signal peptide, *AS* active site sequences coding domains 1 and 2 of the catalytic cleft

Homological primers used for PCR amplification of the gDNA templates produced numerous amplicons of the *CfPAG-L* fragments. Among the 750 electrophoresed, gel-out purified and sequenced *CfPAG-L* gDNA amplicons, 441 clear chromatograms (HQ range: 20–98.2%) were subjected for analyses with GENEIOUS R7 software, which was used to identify 7657 bp of the entire *CfPAG-L* gDNA sequence that have been deposited in the GenBank database (Acc. No. KX377932). Among the identified *CfPAG-L* gDNA sequence, nine exons and eight introns (named A–H) were identified (Fig. 1), as well as donor and acceptor site in exon-intron junctions (Table 2). The sequences at the 5′ donor and 3′ acceptor sites of all introns conformed to the GT-AG rules, and splice junctions were not restricted to any particular phase of a codon (Table 2). Two Asp residues within two domains specific for catalytic cleft of many AP members, predicted previously in *CfPAG-L* cDNA (A. Lipka et al. unpublished), were presently localized within exons 3 and 7 of *CfPAG-L* gDNA (Table 2, Fig. 1).

**Table 2:** Characteristics of exon-intron junctions of the *CfPAG-L* gDNA sequence

Donor splice sites					Acceptor splice sites				
exon	5′- > 3’	Phase	Intron	5′- > 3’	Intron	5′- > 3’	Phase	Exon	5′- > 3’
1	CAATATCCAG	0	A	**GT**GAGCTTGG	A	TGACTTAC**AG**	1	2	TGACTTACAG
2	TTACATGGAT	0	B	**GT**GAGTCCTG	B	CTGCCCCC**AG**	0	3	GCTACTTACT
3	AGCGGCTGCT	1	C	**GT**GAGTGCGG	C	CCCTCTGC**AG**	1	4	CCACACATCC
4	CACTCTGACC	0	D	**GT**AAGTGCAG	D	TCCCCTCC**AG**	1	5	GTCAGAACAT
5	ACCTTGGCAG	2	E	**GT**GAGCATCT	E	TCTCATGC**AG**	2	6	GAAAGAAGGC
6	AAATCGAGGA	2	F	**GT**GAGTTTGT	F	TTCCATTC**AG**	2	7	GTTCCTCGTT
7	AGGAGAACCT	2	G	**GT**TTGGAGAG	G	CTCGCTGC**AG**	0	8	TATTTTGTGA
8	CATCATTAGT	0	H	**GT**AAGTCCTG	H	TGTTGAGC**AG**	0	9	GACGATGGCT

Generally, the determined lengths of the *CfPAG-L* exons (1–9) were similar to exonic lengths of *bPAG1*, *bPAG2*, and *pPAG2*. However, *CfPAG-L* introns (A–H) completely differ compared to previously known *PAG* introns (Table 3). A megablast of the entire *CfPAG-L* gDNA sequence showed some homology only with various BAC clones and pepsinogen C of different species, but 1–5% query cover of these Blast Hits preclude regarding the results as significant. Also, a direct megablast of the *CfPAG-L* with *bPAG1* (Acc. No. AH003454.1) or *pPAG2* (Acc. Nos.: U39198–9; U41421–4; U39762–3; KF471015.1; KF492695.1; KF500427.1; KF527576.1; KF537535.1) revealed no significant similarities. Alignments performed separately for each *CfPAG-L* exon and intron with equivalent segments of *bPAG1* and *pPAG2* structure revealed a lack of homology in the case of intronic regions and 50.3–72.9% homology to exonic regions (Table 4). In silico analyses of the identified *CfPAG-L*-enabled prediction of 31 SNVs (single nucleotide variant), in which 7 were transversions and 24 transitions (Table 5). Among them, 5 identified SNVs were localized within exons and the remaining 26 SNVs within introns. Changes of nucleotide sequence within exons did not affect the amino acid sequence of the CfPAG-L polypeptide precursor, and all identified SNVs were synonymous.

**Table 3:** Nucleotide lengths of the exons and introns of the *CfPAG-L*, *bPAG1*, *bPAG2*, and *pPAG2* gDNA templates

Segment name	*CfPAG-L* [bp]	*bPAG1* [bp]	*bPAG2* [bp]	*pPAG2* [bp]
Exon 1	59	53	53	53
Intron A	1937	1100	1300	1350
Exon 2	160	151	151	166
Intron B	385	1000	1000	1200
Exon 3	118	118	118	118
Intron C	917	100	100	90
Exon 4	119	119	119	119
Intron D	451	1200	1200	1200
Exon 5	200	194	194	200
Intron E	1138	900	1100	850
Exon 6	123	117	117	117
Intron F	288	1900	1700	1800
Exon 7	148	142	142	136
Intron G	681	100	100	85
Exon 8	99	99	99	99
Intron H	603	1700	1700	292
Exon 9	147	150	150	156

**Table 4:** Homology of the *CfPAG-L* exons with equivalents of *bPAG1* and *pPAG2* gDNA

*CfPAG-L*	*bPAG1* [%]	*pPAG2* [%]
Exon 1	60.7	64.3
Exon 2	50.3	60.4
Exon 3	66.1	72.9
Exon 4	56.9	59.2
Exon 5	54.8	60.4
Exon 6	56.9	62.2
Exon 7	55.8	54.2
Exon 8	57	57.4
Exon 9	53.5	56.7

**Table 5:** Identified SNVs, genotypes and allele frequency (px) within the *CfPAG-L* sequence

Type of SNV		Position	Segment	Homozygous						Heterozygous		
				nt	n	p_x_	nt	n	p_x_	nt	n	p_x_
**1**	Transition	109	Intron A	gg	4	0.27	aa	0	0	ga	11	0.73
**2**	Transversion	112	Intron A	tt	4	0.27	gg	0	0	tg	11	0.73
**3**	Transition	2634	Exon 3	gg	0	0	aa	0	0	ga	15	1.0
**4**	Transition	2655	Exon 3	cc	0	0	tt	0	0	ct	15	1.0
**5**	Transition	2668	Intron C	cc	0	0	tt	0	0	ct	15	1.0
**6**	Transition	2671	Intron C	gg	0	0	aa	0	0	ga	15	1.0
**7**	Transition	2676	Intron C	gg	0	0	aa	0	0	ga	15	1.0
**8**	Transition	2702	Intron C	gg	0	0	aa	0	0	ga	15	1.0
**9**	Transition	2721	Intron C	gg	0	0	aa	0	0	ga	15	1.0
**10**	Transition	2738	Intron C	gg	0	0	aa	0	0	ga	15	1.0
**11**	Transition	3803	Intron D	tt	0	0	cc	0	0	tc	15	1.0
**12**	Transition	3822	Intron D	gg	0	0	aa	0	0	ga	15	1.0
**13**	Transition	3890	Intron D	gg	0	0	aa	0	0	ga	15	1.0
**14**	Transition	3912	Intron D	gg	2	0.14	aa	0	0	ga	13	0.86
**15**	Transversion	3936	Intron D	gg	4	0.27	cc	0	0	gc	11	0.73
**16**	Transversion	4098	Intron D	gg	0	0	tt	1	0.07	gt	14	0.93
**17**	Transversion	4124	Intron D	aa	1	0.07	cc	1	0.07	ac	13	0.86
**18**	Transition	4131	Intron D	cc	0	0	tt	0	0	ct	15	1.0
**19**	Transition	4141	Intron D	cc	0	0	tt	0	0	ct	15	1.0
**20**	Transversion	4221	Exon 5	aa	0	0	cc	0	0	ac	15	1.0
**21**	Transversion	6101	Intron G	tt	0	0	gg	0	0	tg	15	1.0
**22**	Transition	6152	Intron G	cc	0	0	tt	0	0	ct	15	1.0
**23**	Transition	6948	Intron H	cc	0	0	tt	0	0	ct	15	1.0
**24**	Transition	6981	Intron H	aa	0	0	gg	0	0	ag	15	1.0
**25**	Transition	6993	Intron H	cc	12	0.8	tt	0	0	ct	3	0.2
**26**	Transversion	7138	Intron H	aa	0	0	tt	0	0	at	15	1.0
**27**	Transition	7140	Intron H	tt	0	0	cc	0	0	tc	15	1.0
**28**	Transition	7345	Intron H	cc	0	0	tt	0	0	ct	15	1.0
**29**	Transition	7406	Intron H	cc	0	0	tt	0	0	ct	15	1.0
**30**	Transition	7537	Exon 9	cc	0	0	tt	0	0	ct	15	1.0
**31**	Transition	7549	Exon 9	cc	0	0	tt	0	0	ct	15	1.0

## Discussion

This is the first study identifying the *PAGs* in the genome of the Eurasian beaver (CfPAG-L). Among the identified 7657 bp of the *CfPAG-L* gDNA sequence, nine exons/eight introns (A–H) and 31 SNVs were found. Generally, the length of the *CfPAG-L* exons (1–9) is similar to exon lengths of *bPAG1*, *bPAG2*, and *pPAG2*. However, the length of the *CfPAG-L* introns (A–H) completely differ from previously known *PAG* introns. The localization of the two Asp residues (D), specific for catalytic cleft of APs, is also conserved in exons 3 and 7 of *CfPAG-L*. Despite the very strong resemblance of the coding region of *CfPAG-L* gene to pepsinogen C (A. Lipka et al. unpublished), there is no such similarity for intronic regions. Additionally, the identified intronic regions of the *CfPAG-L* did not exhibit significant homology to any sequences deposited in GenBank database.

Phylogenetic studies show that the *PAG-L* family arise as a result of a progene duplication or fusion, causing various reproductive capability that may be the result of positive selection of these genes during evolution (Hughes et al. 2003). Lately, limited studies of gDNA revealed the number-diversified presence of the *PAG-L* family in the genomes of some eutherian species, e.g., the elk, yak, wildebeest, impala, and several other antelopes; the pig, goat, horse, cow, sheep, deer, and wild boar and bison (see Szafranska et al. 2006); as well as the alpaca, dromedary, and Bactrian (Majewska et al. 2009). However, unknown exon-intron structure of the *PAGs* has not been studied in the aforementioned and other eutherian species. Previously, based on both *pPAG1* and *pPAG2* cDNA identification (Szafranska et al. 1995), the positions of the exonic and intronic boundaries have been established for the *pPAG2* only (Szafranska et al. 2001). The *pPAG2* represents the first member of the *pPAG2-Like* subfamily (*pPAG2-L*: *pPAG4*, *6*, *8* and *10*) encoding catalytically active APs, although potentially inactive members of the *pPAG1-L* (*pPAG1-L*: *pPAG3* and *5*) have also been identified (Panasiewicz et al. 2004). The *pPAG2* structure (Szafranska et al. 2001), encompasses nine exons (99–200 bp) and eight introns (A–H; 85–1.8 kbp). The shorter introns have been fully sequenced (C, G and H), although the lengths of the longer introns were estimated after PCR amplification and electrophoretic analysis. Recently, nucleotide sequences of the remaining *pPAG2* introns (A, B, D, E and F) have been sequenced and deposited in the GenBank database (KF471015.1; KF492695.1; KF500427.1; KF527576.1; KF537535.1; M. Bieniek-Kobuszewska et al. unpublished). The identified lengths of the introns A, B, D, E, and F are relatively comparable with the predicted ones, according to the *pPAG2* structure (Szafranska et al. 2001). The final length of the *pPAG2* with promoter region is equal to 8755 bp (Bieniek-Kobuszewska et al. 2016). The second known exon-intron organization concerns the *bPAG1* gene (8095 bp) which is similar to other APs and whose intron sizes vary from 87 bp to 1.8 kbp and exon-intron boundaries conform to the standard GT-AG rule for 5′ donor and 3′ acceptor sites (Xie et al. 1995). Moreover, 18 full-length *bPAG* genes with the conserved 9-exon structure of various *PAG-Ls* are represented and properly annotated in the genome assembly (Telugu et al. 2009). Thus, our results are consistent with the exon-intron structures of three known *PAG* genes: *bPAG1* (Xie et al. 1995), *pPAG2* (Szafranska et al. 2001) and *bPAG2* (Telugu et al. 2009).

The obtained results regarding SNVs within *CfPAG-L* gene may constitute a basis for further genome-wide association (GWA) studies (Appels et al. 2013; Akpinar et al. 2017). The relationship between a specific genotype and a phenotype can be used to predict genes that may correlate with observable traits in various animals. Presently, since our SNV data of the *CfPAG-L* cannot be compared in the beavers because such data are unavailable, we will therefore discuss the data in relation to the *PAGs* in other species. Previously, 32 SNPs/InDel and 42 SNPs/InDels have been identified within proximal and flanking distal regions of the *pPAG2-L* promoter in cross-breed pigs and in the Duroc breed, respectively (Bieniek-Kobuszewska et al. 2016). Many of those SNPs have been identified within transcription factor binding sites, which suggests the importance of allelic diversity and the significant influence on regulation of the *pPAG2-L* expression. Other studies concerning polymorphism in *pPAG2-L* gene indicate that the SNPs identified within the exon 6 and the intron F are associated with reproductive traits, i.e., the number of the born alive and weaned piglets (G. Panasiewicz et al. unpublished). Thus, in the case of our results it cannot be excluded that predicted SNVs may be involved in positive or negative regulation of placenta development, which may seriously affect pregnancy outcome, as it was previously suggested in human (Majewska et al. 2017). To date, GWA studies have been mainly focused on the selection of domestic animals with the best traits for breeding (Hering et al. 2014). In the future, our results may be useful to establish a genetic marker for the selection of unrelated representatives of various endangered animals for reconstruction or reintroduction programs.

Finally, since the beaver is not a common object of research it is extremely difficult to compare and discuss the obtained genetic results. However, considering the direct impact of this species on the environment and its significant influence on all other organisms inhabiting that environment, we are confident that the beaver will become a more common object of biological and economic interest.

This study provided pioneering data on the *CfPAG-L* family in the genome of the beaver, the largest rodent in Europe. Our data will presumably have an influence on further explanation of proper genetic regulation, efficient implantation and pregnancy maintenance in this species. Our results extend present knowledge about the beaver genome, and in the future, will help to improve the possibility of biodiversity conservation and genetic resource protection in Poland and other countries.
